# Genetically engineered mouse models in oncology research and cancer medicine

**DOI:** 10.15252/emmm.201606857

**Published:** 2016-12-27

**Authors:** Kelly Kersten, Karin E de Visser, Martine H van Miltenburg, Jos Jonkers

**Affiliations:** ^1^ Division of ImmunologyThe Netherlands Cancer InstituteAmsterdamThe Netherlands; ^2^ Division of Molecular Pathology and Cancer Genomics NetherlandsThe Netherlands Cancer InstituteAmsterdamThe Netherlands

**Keywords:** cancer, genetically engineered mouse models, metastasis, therapy, tumor microenvironment, Cancer

## Abstract

Genetically engineered mouse models (GEMMs) have contributed significantly to the field of cancer research. In contrast to cancer cell inoculation models, GEMMs develop *de novo* tumors in a natural immune‐proficient microenvironment. Tumors arising in advanced GEMMs closely mimic the histopathological and molecular features of their human counterparts, display genetic heterogeneity, and are able to spontaneously progress toward metastatic disease. As such, GEMMs are generally superior to cancer cell inoculation models, which show no or limited heterogeneity and are often metastatic from the start. Given that GEMMs capture both tumor cell‐intrinsic and cell‐extrinsic factors that drive *de novo* tumor initiation and progression toward metastatic disease, these models are indispensable for preclinical research. GEMMs have successfully been used to validate candidate cancer genes and drug targets, assess therapy efficacy, dissect the impact of the tumor microenvironment, and evaluate mechanisms of drug resistance. *In vivo* validation of candidate cancer genes and therapeutic targets is further accelerated by recent advances in genetic engineering that enable fast‐track generation and fine‐tuning of GEMMs to more closely resemble human patients. In addition, aligning preclinical tumor intervention studies in advanced GEMMs with clinical studies in patients is expected to accelerate the development of novel therapeutic strategies and their translation into the clinic.

**Glossary**
Allotransplantation: Transplantation of established mouse cancer cell lines in immunoproficient mice with the same genetic background.
Cancer immunotherapy: Therapeutic strategies aimed at harnessing the patient's immune system to attack cancer. For example, immune checkpoint blockade interferes with negative regulatory molecules of T‐cell activation.
Cancer‐associated fibroblasts (CAFs): Fibroblasts that reside in the tumor microenvironment.
Clinically overt metastatic disease: Metastatic disease that affects multiple organs, eventually leading to organ failure and death.
Conventional GEMM: Oncogene‐bearing transgenic mice (aka oncomice) and mice carrying germline mutations in tumor suppressor genes (TSGs).
Cre‐ERT: A fusion protein in which Cre‐recombinase is fused to a mutated hormone‐binding domain of the estrogen receptor (ERT). Administration of the estrogen analogue tamoxifen leads to post‐translational activation of Cre‐recombinase activity and excision of the target gene flanked by *loxP* sites.
Cre‐*loxP*: A site‐specific recombination system that allows for Cre‐recombinase‐mediated deletion of genes flanked by *loxP* recombination sites. Expression of Cre‐recombinase can be induced in a tissue‐restricted manner.
CRISPR/Cas9: A genome editing system that enables induction of DNA double‐strand breaks (DSBs) at defined genomic locations by directing the Cas9 nuclease to a predefined genomic locus using single‐guide RNAs (sgRNAs). DSB repair by non‐homologous end‐joining or homologous recombination (in the presence of an oligonucleotide) will lead to gene knockout or modification, respectively.
Epithelial‐to‐mesenchymal transition (EMT): A process by which epithelial cells lose polarity and cell–cell adhesion, and gain mesenchymal‐like migratory properties.
Extracellular matrix (ECM): The non‐cellular component present within all tissues and organs, which provides essential physical scaffolding for cellular structures and has biochemical and mechanical properties important for tissue development and homeostasis.
Flp‐*FRT*: A site‐specific recombination system that allows for Flp‐recombinase‐mediated deletion of genes flanked by *FRT* recombination sites.
GEMM‐ESC: A technique for rapid introduction of additional genetic modifications and subsequent production of chimeric mice from embryonic stem cells (ESCs) derived from existing GEMMs.
Germline GEMMs: Mouse models carrying genetically engineered alleles (transgenes, conventional knockout/knock‐in alleles, or loxP/FRT‐flanked conditional alleles) in all cells including the germline.
Next‐generation GEMMs: Mouse models that are genetically engineered to accurately mimic sporadic human cancer.
Non‐germline GEMMs: Mouse models carrying genetically engineered alleles in somatic cells but not in germline cells. These mouse models include chimeric mice derived from genetically engineered (GEMM‐derived) ESCs and mice produced by CRISPR/Cas9‐mediated somatic gene editing.
Oncogene addiction: Tumors display “oncogene addiction” when they are highly dependent on a single oncogene for their growth and maintenance.
Oncomouse: Mouse with transgenic expression of a specific oncogene under control of a tissue‐specific promoter.
Patient‐derived tumor xenografts (PDTX): Mouse models based on transplantation and serial propagation of fresh human tumor biopsies in immunodeficient mice.
Tumor microenvironment (TME): The cellular environment in which tumor cells reside. The tumor microenvironment is composed of different populations of stromal cells, including endothelial cells, fibroblasts, extracellular matrix, and immune cells.
Xenotransplantation: Transplantation of human tumor cells or tissue in immunocom‐promised mice.

## Introduction

Despite the fact that survival rates of cancer patients have improved over the last decades, we are still facing numerous challenges in the clinic. One of the major problems is the development of drug resistance. Monotherapy with targeted anti‐cancer agents or chemotherapeutics invariably results in drug resistance caused by *de novo* mutations or outgrowth of pre‐existing therapy‐resistant clones within heterogeneous tumors. Moreover, after apparently successful treatments, small numbers of drug‐tolerant tumor cells can survive and remain dormant for extended periods of time and eventually relapse to form recurrent disease that can be phenotypically different from the original tumor (Kottke *et al*, 2013; Blatter & Rottenberg, 2015). Another major challenge is metastatic disease, which accounts for over ninety percent of cancer‐related deaths (Weigelt *et al*, 2005). These secondary tumors are often unresponsive to therapy and are at present mostly incurable. Encouraging advancements have been made with cancer immunotherapy, aimed at harnessing the patient's immune system to attack cancer. However, even though durable responses are observed in some cases, a large proportion of cancer patients does not show clinical benefit (Sharma & Allison, 2015).

**Figure 1:**
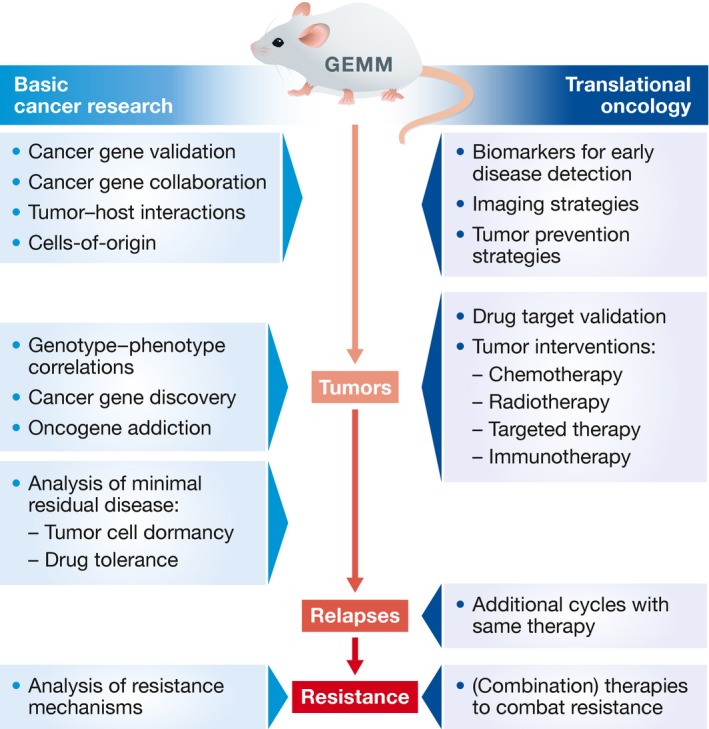
Applications of GEMMs in basic cancer research and translational oncology

Successful treatment of cancer requires a multidisciplinary approach in which different strategies such as surgery, irradiation, cytotoxic therapy, and immunotherapy are combined. To design such combinations, it is critical to improve our insights into the cancer cell‐intrinsic and cell‐extrinsic mechanisms underlying tumor development, metastasis, and therapy responsiveness. To find the most effective treatment for different cancer types, we heavily rely on preclinical research in animal models. Despite successful validation of novel anti‐cancer therapies in conventional preclinical mouse models based on xenotransplantation of established human cancer cell lines or allotransplantation of mouse tumor cell lines, the majority of the phase 3 clinical trials fail (Reichert & Wenger, 2008). The overall poor clinical predictability of these conventional *in vivo* tumor models emphasizes the need for more advanced preclinical *in vivo* models with better predictive power. Until fairly recently, progress in the field was hampered by the poor availability of preclinical models that closely recapitulate the natural course of human cancer. However, recent technological developments have led to fast‐track generation of sophisticated mouse models that more closely mimic human cancer in terms of genetic composition, interactions of cancer cells with their tumor microenvironment, drug response, and resistance. These next‐generation genetically engineered mouse models (GEMMs) are of great importance to improve our understanding of the complex mechanisms underlying cancer biology, and are anticipated to improve translation of new therapeutic strategies into the clinic (Fig 1)—ultimately leading to increased survival of cancer patients. This review describes the evolution and recent technological advances of mouse model engineering, and the applications of the resulting models in basic and translational oncology research.

## Evolution of mouse cancer modeling

**Figure 2:**
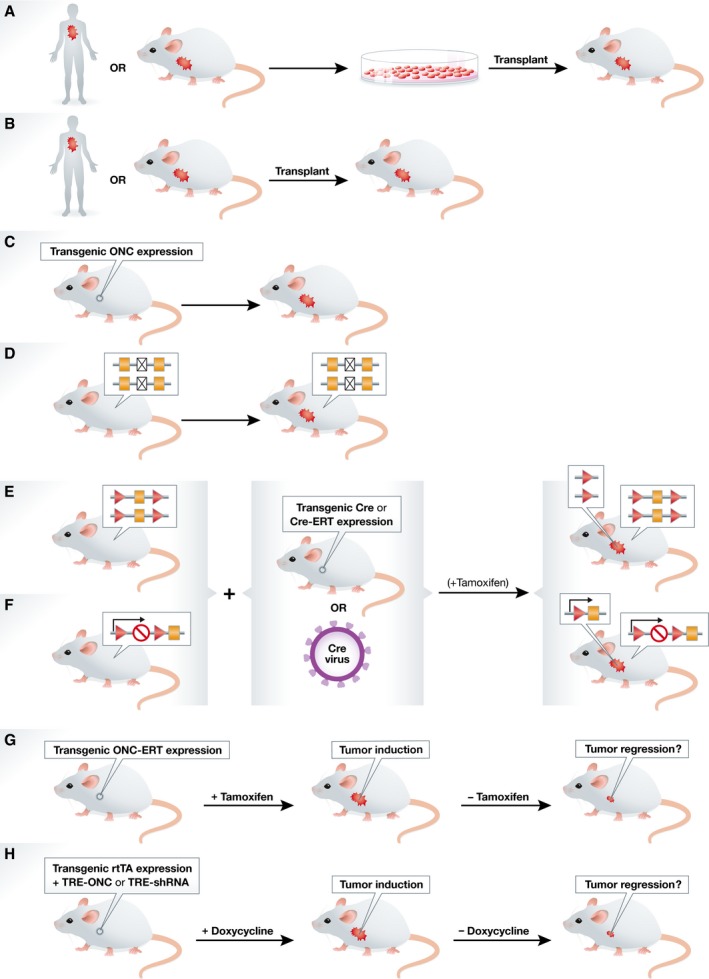
Schematic overview of transplantation‐based mouse models and germline GEMMs (A) Cancer cell line transplantation models are based on (orthotopic) inoculation of cultured human or mouse cancer cells in immunodeficient or syngeneic mice, respectively. (B) Patient‐derived tumor xenograft models or GEMM‐derived tumor allograft models are based on direct (orthotopic) implantation of human or mouse tumor fragments in immunodeficient or syngeneic mice, respectively. (C) In oncomice, *de novo* tumorigenesis is induced by transgenic expression of an oncogene (ONC) from a tissue‐specific promoter. (D) In tumor suppressor gene (TSG) knockout mice, *de novo* tumorigenesis is induced by germline inactivation of a TSG. (E, F) In conditional GEMMs, *de novo* formation of sporadic tumors is induced by tissue‐specific Cre‐*loxP‐*mediated inactivation of conditional TSG alleles (E) and/or activation of conditional oncogenes (F). Tissue‐specific expression of the Cre‐recombinase is achieved by crossbreeding with Cre transgenic mice, tamoxifen‐inducible Cre‐ERT transgenic mice, or by local administration of Cre‐encoding lenti‐ or adenoviruses. (G, H) Oncogene addiction can be studied using GEMMs with tamoxifen‐ or doxycycline‐inducible gene expression. (G) Administration of tamoxifen to transgenic mice carrying an oncogene‐ERT (ONC‐ERT) fusion will induce tumors that may undergo no, temporal, or durable regression upon tamoxifen withdrawal. (H) Similar studies can be performed by administration of doxycycline to bi‐transgenic mice with tissue‐specific expression of the reverse tetracycline‐controlled transactivator (rtTA) and carrying an oncogene or shRNA under transcriptional control of a tetracycline response element (TRE).

Since the advent of tumor transplantation models in nude mice 50 years ago, advances in mouse genome engineering have led to the generation of various types of transplantation‐based and genetically engineered tumor models to study cancer biology (Fig 2, Table 1). Here, we give an overview of mouse models that are most commonly used in cancer research.

**Table 1:** Advantages and disadvantages of different mouse cancer models

	*De novo* carcinogenesis	Natural immunocompetent TME	Spontaneous metastasis	Cancer gene validation	Genetic validation drug targets	Preclinical drug testing	Drug resistance mechanisms	Immunotherapy
Allograft cell line inoculation	No	Maybe	Model‐dependent	Yes	Yes	Yes	Yes	Yes
Xenograft cell line inoculation^a^	No	No	Model‐dependent	Yes	Yes	Yes	Maybe	No
Patient‐derived tumor xenograft (PDTX)^a^	No	No	Model‐dependent	No	No	Yes	Yes	No
Oncomouse	Yes	Yes	Model‐dependent	Yes	No	Yes	Yes	Yes
Cre‐*LoxP*	Yes	Yes	Model‐dependent	Yes	No	Yes	Yes	Yes
Tet‐on/off TetO‐Cre	Yes	Yes	Model‐dependent	Yes	Yes	Yes	Yes	Yes
GEMM‐ESC	Yes	Yes	Model‐dependent	Yes	Yes	Yes	Yes	Yes
CRISPR/Cas9	Yes	Yes, when used in Cas9‐tolerant hosts	Model‐dependent	Yes	Yes	Yes	Yes	Expected

a These cancer models require the use of immunodeficient hosts.

### Cancer cell line transplantation models

Mouse models based on xenografting human cancer cell lines or allografting mouse tumor cells are the most commonly used *in vivo* tumor models in cancer research. These transplantation models allow for rapid testing of potential cancer‐ and metastasis‐related genes and are often used for preclinical drug testing. For example, xenotransplantation studies provided insights into the mechanisms underlying intrinsic resistance of colorectal cancer (CRC) to vemurafenib. The results of this study led to the initiation of a clinical trial in which CRC patients were treated with a combination therapy targeting both *BRAF* ^*V600E*^ and EGFR (Prahallad *et al*, 2012), illustrating the usefulness of xenotransplantation models in establishing novel combinatorial treatment strategies. Moreover, xenograft studies identified distinct gene expression signatures that mediate organ‐specific patterns of metastatic colonization (Kang *et al*, 2003; Minn *et al*, 2005; Bos *et al*, 2009). In addition, these models revealed that disseminated breast cancer cells reside adjacent to blood vessels, which might provide a niche regulating dormancy of disseminated cancer cells (Ghajar *et al*, 2013). Furthermore, many of the fundamental insights into anti‐tumor immunity, T‐cell tolerance mechanisms, and immune‐escape routes of tumors come from *in vivo* studies with cell line allograft models (Leach *et al*, 1996). These findings have laid the foundation for the currently ongoing cancer immunotherapy revolution.

Nevertheless, as cancer cell lines contain multiple mutations from the start and acquire additional aberrations when cultured *in vitro* for extended periods of time, these inoculation models do not reflect the morphology and genetic heterogeneity of human cancers, and are therefore mostly poor predictors of clinical response. While allografting of mouse cancer cell lines can be performed in immunoproficient hosts, xenotransplantation of cell lines must be performed in immunocompromised mice to prevent rejection, which makes them less suitable to study the roles of the immune system in tumor development and therapy response.

### Patient‐derived tumor xenografts

Patient‐derived tumor xenografts (PDTX) are derived from fresh human tumor biopsies that are transplanted in immunodeficient mice. Unlike cell line transplantation models, PDTX tumors maintain the molecular, genetic, and histological heterogeneity as observed in cancer patients, even after serial passaging in mice (Hidalgo *et al*, 2014). Therefore, PDTX models can be valuable tools to define personalized medicine as was demonstrated by preclinical drug screening in PDTX models of non‐small‐cell lung cancer (NSCLC) (Fichtner *et al*, 2008; Merk *et al*, 2009, 2011), breast cancer (Marangoni *et al*, 2007), melanoma (Kemper *et al*, 2015; Girotti *et al*, 2016), prostate cancer (Yoshida *et al*, 2005; Qu *et al*, 2014), and colorectal cancer (Bertotti *et al*, 2011, 2015; Bardelli *et al*, 2013; Kavuri *et al*, 2015; Misale *et al*, 2015; Zanella *et al*, 2015). For small‐cell lung cancer (SCLC), it was shown that circulating tumor cells (CTCs) from blood can be used to establish CTC‐derived explant (CDX) models that mirror the donor patient's response to platinum and etoposide (Hodgkinson *et al*, 2014). Large‐scale studies are now carried out using PDTX models to predict responses of clinical drug candidates. Approximately 1,000 PDTX models were established with a diverse panel of mutations, and subsequently used for *in vivo* compound screens, yielding correlations between drug response and tumor genotype that were both reproducible and clinically translatable (Gao *et al*, 2015). In a recent study using PDTX models of triple‐negative breast cancer, single‐cell gene expression analysis revealed that early‐stage metastatic cells express distinct signatures enriched in stem‐like genes, identifying novel potential drug targets to tackle metastatic breast cancer (Lawson *et al*, 2015).

Unfortunately, a major obstacle of PDTX modeling is the disappointing take rate of various tumor types, such as estrogen receptor‐positive breast cancer and prostate cancer (Landis *et al*, 2013; Lawrence *et al*, 2015). In addition, PDTX modeling must be performed in immunocompromised mice, thereby circumventing the natural anti‐ and pro‐tumor activity provided by the adaptive immune system. Given the complex crosstalk between adaptive immune components, the innate immune system, and cancer cells, it is important to realize that PDTX models can provide clinically valuable data, albeit in the absence of the influential adaptive immune system. Current efforts to generate humanized mice by engrafting immunodeficient mice with human CD34^+^ hematopoietic stem cells or precursor cells have shown remarkable progress (Drake *et al*, 2012; Holzapfel *et al*, 2015). Although reconstitution of immune cells from specific lineages remains challenging, the introduction of transgenes encoding human cytokines, chemokines, and growth factors can support the development of human myeloid cells in mice. To support development of HLA‐restricted T cells, recipient immunodeficient mice can be further optimized by transgenic expression of human HLA molecules and deficiency of mouse MHC class‐I and class‐II molecules. While the limited availability of hematopoietic donor stem cells (obtained from umbilical cord blood or fetal liver) and the relatively high costs of these models are potential disadvantages, humanized mouse models could provide a useful platform for preclinical evaluation of immunotherapeutics.

### Modeling de novo cancer in genetically engineered mice

In the early 1980s, the first cloned cancer genes were introduced into the genome of transgenic mice, which were termed oncomice (Hanahan *et al*, 2007). The first oncomouse was a GEMM with transgenic expression of a specific activated oncogene (*v‐HRas*) under control of a mammary‐specific promoter (*MMTV*), making the mouse prone to developing mammary tumors (Sinn *et al*, 1987). The first oncomice led to great excitement in the cancer research community as they provided unambiguous proof for the hypothesis that oncogene expression in normal cells could lead to tumor formation (Brinster *et al*, 1984; Hanahan, 1985; Lacey *et al*, 1986; Sinn *et al*, 1987; Rüther *et al*, 1989). With the development of gene‐targeting technology in 1992, also cancer predisposition in tumor suppressor gene (TSG) knockout mice could be studied (Finlay, 1992).

Though oncomice and TSG knockout mice have provided a wealth of knowledge, they also have their limitations. Given that transgenes are expressed in all cells of a particular tissue and TSGs in knockout mice are inactivated in all cells of the animal, these models fail to mimic sporadic cancers in which accumulation of genetic events in a single cell results in tumorigenesis in an otherwise healthy organ. To circumvent this, more sophisticated mouse models are currently available that allow somatic inactivation of tumor suppressors or activation of (mutant) oncogenes in conditional GEMMs (Jonkers & Berns, 2002). One of the first examples is the generation of a mouse colorectal cancer model using Cre‐*loxP*‐mediated somatic inactivation of *Apc*. With this technique, any gene flanked by *loxP* recombination sites will be deleted after activation of the Cre‐recombinase. APC loss in intestinal epithelial cells was sporadically induced through adenovirus‐mediated delivery of Cre‐recombinase, resulting in the rapid onset of colorectal adenomas that shared many features with adenomas in familial adenomatous polyposis coli (FAP) patients (Shibata *et al*, 1997). By introducing mutations associated with a specific type of cancer, one can generate mouse models that closely mimic the histopathological, molecular, and clinical features of tumors in patients (Frese & Tuveson, 2007; Walrath *et al*, 2010).

Induction of somatic mutations at a chosen time and in a specific tissue can be achieved by using Cre‐ERT fusion proteins, in which a mutated hormone‐binding domain of the estrogen receptor (ERT) is fused to the Cre‐recombinase. Cre‐ERT is an inducible Cre‐recombinase: administration of the estrogen analogue tamoxifen leads to post‐translational activation of Cre‐recombinase activity and excision of the target gene flanked by *loxP* sites. Hence, mice with (tissue‐specific) expression of Cre‐ERT allow for spatiotemporally controlled Cre‐mediated genomic recombination upon administration of tamoxifen (Vooijs *et al*, 2001).

Even though the Cre‐*loxP* system can be applied to alter the expression of more than one gene, it does so simultaneously, and therefore does not fully mimic the sequential accumulation of mutations during multistep carcinogenesis. Recently, an inducible dual‐recombinase system was developed which combines Flp‐*FRT* and Cre‐*loxP* recombination systems, allowing sequential genetic manipulation of gene expression by two independent recombination systems (Schönhuber *et al*, 2014). This approach allows for (i) independent targeting of tumor cell autonomous and non‐autonomous pathways/processes, (ii) sequential induction of mutations to model human multistep carcinogenesis, and (iii) genetic validation of therapeutic targets in autochthonous tumors.

## Speeding up and fine‐tuning mouse cancer modeling

**Figure 3:**
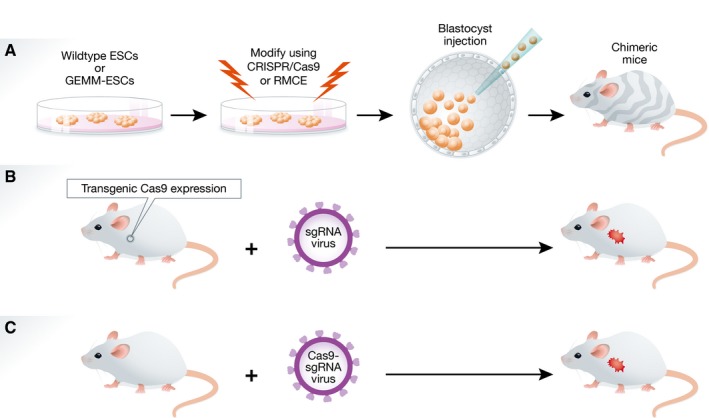
Schematic overview of non‐germline GEMMs (A) Embryonic stem cell (ESC)‐derived non‐germline GEMMs. ESCs from wild‐type mice or established GEMMs can be used to introduce single or multiple mutations using CRISPR/Cas9‐based gene editing and/or mutant alleles using recombinase‐mediated cassette exchange (RMCE). The resulting ESCs can be injected into host blastocysts, which are implanted into pseudo‐pregnant females to produce chimeric mice. (B, C) CRISPR/Cas9‐based non‐germline GEMMs. CRISPR/Cas9‐mediated *in situ* gene editing can be achieved by local administration of sgRNA‐encoding lentiviruses in transgenic mice with tissue‐specific Cas9 expression (B), or by local administration of lentiviruses that encode both Cas9 and sgRNA in wild‐type mice (C). The latter approach may require immunodeficient or Cas9‐tolerant mice to avoid Cas9‐specific immune responses.

While GEMMs have proven to be valuable tools for cancer research, there are still aspects that can be improved. A major limitation of germline GEMMs is that development and validation of these models is time‐consuming, laborious, and expensive. This is exemplified when a novel germline mutation has to be introduced in an existing multi‐allelic mouse model, as this requires extensive breeding. The rapidly increasing number of mutations identified in cancer sequencing studies calls for novel mouse modeling strategies that enable accelerated *in vivo* evaluation of candidate cancer genes and patient‐relevant mutations in known cancer genes in non‐germline GEMMs (Fig 3).

### Embryonic stem cell‐based mouse cancer models

To speed up the generation of novel GEMMs of human cancer, embryonic stem cells (ESCs) can be genetically altered and used to produce cohorts of non‐germline GEMMs (Heyer *et al*, 2010). An alternative approach is the recently developed GEMM‐ESC strategy, which employs ESCs that are derived from existing (multi‐allelic) GEMMs. These GEMM‐derived ESCs can be used for rapid introduction of additional genetic modifications and subsequent production of chimeric mice that show the same characteristics as the established GEMM but now contain the additional genetic modification (Huijbers *et al*, 2011, 2014). For example, the GEMM‐ESC strategy was used to introduce the *Met* proto‐oncogene in a GEMM of BRCA1‐associated breast cancer, which yielded a novel mouse model of BRCA1‐deficient metaplastic breast cancer (Henneman *et al*, 2015). Whereas BRCA1‐deficient mouse mammary carcinomas showed high sensitivity to the clinical PARP inhibitor olaparib, BRCA1‐deficient metaplastic mammary tumors showed intrinsic resistance.

### In vivo RNA interference

RNA interference (RNAi) by short hairpin RNAs (shRNAs) allows reversible silencing of gene expression without modifying the genome, and therefore, it can be used as an alternative to homologous recombination‐based gene inactivation approaches. RNAi‐based genetic screens have proven to be powerful tools to rapidly identify and validate cancer genes. *In vivo* RNAi screens have been successfully used to identify novel TSGs in mouse models of hepatocellular carcinoma and lymphoma (Hemann *et al*, 2003; Zender *et al*, 2008; Bric *et al*, 2009), and to identify genes involved in resistance to the tyrosine kinase inhibitor sorafenib in liver cancer (Rudalska *et al*, 2014). Moreover, the development of systems for doxycycline‐inducible shRNA expression in transgenic mice allows reversible expression of shRNAs in a time‐ and tissue‐specific manner (Dickins *et al*, 2007; Premsrirut *et al*, 2011). Using the latter approach, Dow *et al* (2015b) have shown that shRNA‐mediated APC suppression in the presence of *Kras* and *Trp53* mutations induces intestinal carcinomas, which undergo sustained regression upon restoration of APC expression by turning off shRNA expression.

### Genome editing using CRISPR/Cas9 technology

In the past decades, additional approaches for genome editing have been developed such as Zinc‐finger nucleases (ZFNs) and transcription‐activator‐like effector nucleases (TALENs) (Li *et al*, 2011; Wefers *et al*, 2013). These approaches have now been outperformed by the development of CRISPR/Cas9 systems for genome editing (Cong *et al*, 2013), which have revolutionized biological research over the past 3 years and are considered the biggest game changer since PCR. The CRISPR (clustered regularly interspaced short palindromic repeats)/Cas9 system was first discovered as a prokaryotic immune system that confers resistance to foreign genetic elements, but soon thereafter has been exploited to achieve gene editing (Ishino *et al*, 1987; Mojica *et al*, 2000; Jansen *et al*, 2002). By using appropriate single‐guide RNAs (sgRNAs), the Cas9 nuclease can be directed to any genomic locus, where it induces double‐stranded cleavage of matching target DNA sequences, leading to gene knockout (Cong *et al*, 2013). The CRISPR/Cas9 system can also be used to introduce defined mutations or *loxP*/*FRT* recombination sites, by simply co‐introducing oligonucleotides that can serve as a template for repair of the Cas9‐induced break (Yang *et al*, 2014).

CRISPR/Cas9 technology seems the system of choice for rapid cancer modeling in mice, as it has proven to be an efficient gene‐targeting strategy with the potential for multiplexed genome editing (Sánchez‐Rivera & Jacks, 2015). Virtually, all (combinations of) genetic alterations found in human tumors can now be rapidly introduced in the mouse germline, including (conditional) gene deletions (Wang *et al*, 2013; Yang *et al*, 2013), point mutations (Wang *et al*, 2013), and translocations (Blasco *et al*, 2014; Choi & Meyerson, 2014; Torres *et al*, 2014). Other groups have successfully used CRISPR/Cas9 technology for somatic editing of oncogenes and TSGs in mice. These efforts have led to a new generation of non‐germline models of hepatocellular carcinoma (Xue *et al*, 2014; Weber *et al*, 2015), lung cancer (Platt *et al*, 2014; Sánchez‐Rivera *et al*, 2014), brain cancer (Zuckermann *et al*, 2015), pancreatic cancer (Chiou *et al*, 2015; Maresch *et al*, 2016), and breast cancer (Annunziato *et al*, 2016).

The CRISPR/Cas9 system has recently been modified to induce target gene repression (CRISPRi) or activation (CRISPRa) (Gilbert *et al*, 2013). These modified systems may be used to generate mice with inducible and reversible activation of oncogenes and/or inactivation of TSGs. Though extremely powerful, CRISPR/Cas9‐based systems for *in vivo* gene editing may also have certain drawbacks. For example, current CRISPR/Cas9 strategies are not suited to validate the oncogenic potential of putative oncogenes. To this end, CRISPRa‐based systems may be used to activate transcription of target genes (Braun *et al*, 2016). Moreover, somatic delivery of Cas9 may trigger Cas9‐specific immune responses resulting in clearance of Cas9‐expressing cells (Wang *et al*, 2015; Annunziato *et al*, 2016). To circumvent this issue, experiments should be performed in immunodeficient animals or mice that are engineered to develop immunological tolerance to Cas9. Finally, CRISPR/Cas9‐mediated genome editing may create unwanted off‐target mutations that may be circumvented by employing pairs of sgRNAs in mice with inducible expression of a Cas9n “nickase” variant that induces DNA single‐strand breaks (Dow *et al*, 2015a).

### Fine‐tuning mouse cancer modeling with patient‐relevant alleles

Many cancer‐predisposing germline mutations and somatic mutations in human TSGs are missense or nonsense mutations that may result in the production of a mutant or truncated protein with residual activity. Such mutations are not adequately modeled in (conditional) knockout mice, in which deletion of one or more exons leads to complete loss of the protein. It is therefore essential to generate mouse models carrying patient‐relevant mutations to study their contribution to tumorigenesis and therapy response. Several studies have shown that patient‐relevant TSG mutations in mice induce different phenotypes compared to the null‐alleles. Compared to *Trp53* knockouts, patient‐relevant *Trp53* hotspot mutations in mice were shown to have enhanced oncogenic activity (Lang *et al*, 2004; Olive *et al*, 2004). Similarly, introduction of patient‐relevant *Brca1* mutations in a conditional mouse model of BRCA1‐associated breast cancer showed that, in contrast to *Brca1*‐null tumors, mammary tumors with expression of *Brca1* alleles harboring mutations in the RING domain readily acquired resistance to DNA‐damaging drugs due to residual activity of the RING‐less BRCA1 protein (Drost *et al*, 2011, 2016). Thus, by introducing specific somatic or germline mutations into GEMMs, the causal link between these mutations and therapy responsiveness can be determined.

## Applications of GEMMs in basic cancer research

GEMMs of *de novo* tumorigenesis are the systems of choice for *in vivo* analysis of the cell‐intrinsic and cell‐extrinsic processes that contribute to cancer initiation, progression, and metastasis. Here, we discuss how GEMMs have contributed to advances in cancer biology.

### Validation of candidate cancer genes

Given the growing number of candidate cancer genes that are identified in large‐scale tumor sequencing studies, there is a clear need for rapid *in vivo* strategies to validate these genes. Considering their speed and relative simplicity, GEMM‐ESC and CRISPR/Cas9 technologies are the methods of choice for fast‐track validation of candidate cancer genes. Especially, non‐germline models based on somatic CRISPR/Cas9‐mediated gene editing enable *in vivo* validation of (combinations of) candidate cancer genes in a truly high‐throughput manner, as was demonstrated in a mouse model for pancreatic cancer (Maresch *et al*, 2016). Here, transfection‐based multiplexed delivery of Cas9 and sgRNAs targeting 13 different cancer genes induced pancreatic cancer (PDAC) in the majority of mice. The PDACs displayed genome editing of over 60% of the target genes, indicating clonal expansion of CRISPR/Cas9‐induced driver mutations that induce cancer (Maresch *et al*, 2016). Likewise, GEMMs with doxycycline‐inducible Cas9 expression were employed to validate defined combinations of intestinal cancer genes, for example, *Apc* and *Trp53* (Dow *et al*, 2015a). Besides modifying TSGs, CRISPR/Cas9 technology can be applied to validate the oncogenicity of chromosomal rearrangements, such as the *Eml4‐Alk* gene fusion observed in lung cancer (Maddalo *et al*, 2014).

### Mouse models to study oncogene addiction

Some tumors are highly dependent on a single oncogene for their growth, a phenomenon called “oncogene addiction”. Conditional GEMMs are unsuitable models to determine oncogene addiction, as the genetic lesion is irreversible, and thus requires another layer of regulation. Oncogene‐ERT fusions can be employed to control oncogene expression; for example, *Trp53* ^*KI*/*KI*^ mice in which both *Trp53* alleles are replaced by a tamoxifen‐inducible *Trp53‐ERT* variant and were used to determine the therapeutic efficacy of p53 restoration in established tumors (Martins *et al*, 2006). P53 restoration in established *Eμ‐Myc* lymphomas triggered rapid apoptosis, which led to a significant increase in survival.

Also systems for doxycycline‐regulatable gene expression have been successfully used in GEMMs to turn oncogenes on to induce tumorigenesis; and off to determine how established tumors respond to oncogene inactivation (Gossen *et al*, 1995; Lewandoski, 2001). For example, continuous expression of a doxycycline‐inducible *Myc* transgene in hematopoietic cells resulted in the formation of malignant T‐cell lymphomas and acute myeloid leukemia that regressed upon de‐induction of *Myc* expression (Felsher & Bishop, 1999). The long‐term effects of temporal MYC de‐induction seem to differ between cancer types. For example, brief inactivation of MYC in osteogenic sarcomas resulted in sustained regression due to differentiation of sarcoma cells into mature osteocytes (Jain *et al*, 2002). In contrast, invasive liver cancers regressed after MYC inactivation, but residual tumor cells remained dormant and immediately restored their neoplastic features upon MYC reactivation (Shachaf *et al*, 2004).

### Determining cells‐of‐origin of cancers

Identifying the cell‐of‐origin of malignancies may provide important information for the development of improved therapeutic strategies. Studies in GEMMs have successfully identified the cell‐of‐origin for several different cancer types. For example, the cell‐of‐origin of SCLC was determined by intratracheal injection of cell‐type‐restricted Adeno‐Cre viruses, to inactivate *Trp53* and *Rb1* in Clara, neuro‐endocrine (NE), and alveolar type 2 (SPC) cells, respectively. *Trp53* and *Rb1* inactivation in these specific cell types of the lung resulted in differences in tumor onset and tumor phenotype, and identified NE cells (and to a lesser extent SPC cells) as the cell‐of‐origin in SCLC (Sutherland *et al*, 2011). Cell‐of‐origin studies can also deliver surprising results, as was the case for BRCA1‐related basal‐like breast cancer. While BRCA1‐related basal‐like breast cancer was previously postulated to originate from basal epithelial stem cells, cell‐of‐origin studies in GEMMs revealed that in fact, luminal progenitors are the source of basal‐like tumors (Molyneux *et al*, 2010). Genetic aberrations, such as *Pik3ca* mutations, can have a profound effect on the stem cell pool, as was demonstrated recently by two independent laboratories. Expression of *Pik3ca* ^*H1047R*^ was shown to evoke dedifferentiation of lineage‐committed mammary epithelial cells into a multipotent stem‐like state (Koren *et al*, 2015; Van Keymeulen *et al*, 2015). Interestingly, the cell‐of‐origin of *Pik3ca* ^*H1047R*^ mammary tumors dictates their malignancy, highlighting the importance of pinpointing the cell‐of‐origin to improve specificity of anti‐cancer drugs and therapeutic outcome.

### Studying the contribution of the tumor microenvironment

GEMMs have been indispensable in deciphering the contribution of tumor cell‐extrinsic factors such as cancer‐associated fibroblasts (CAFs) and immune cells to tumorigenesis. CAFs regulate deposition of extracellular matrix (ECM) and formation of basement membrane by synthesizing ECM components such as collagen, fibronectin, and laminin. Moreover, fibroblasts are a source of various soluble mediators including matrix metalloproteinases (MMPs), which enable ECM turnover, reinforcing their crucial role in maintaining ECM homeostasis (Kalluri & Zeisberg, 2006). Studies in GEMMs have demonstrated dual roles of fibroblasts in cancer. During malignant transformation of epithelial cells, CAFs can stimulate tumor progression by enhancing inflammation, angiogenesis, and ECM remodeling, as was demonstrated in the *K14‐HPV16* squamous skin cancer model (Erez *et al*, 2010). In contrast, genetic *in vivo* depletion of CAFs was shown to accelerate tumor progression in two independent GEMMs of pancreatic cancer (Özdemir *et al*, 2014), suggesting a tumor‐restraining role for CAFs. The same controversy holds true for immune cells: originally, immune cells were thought to suppress tumorigenesis by attacking transformed cells; however, recent studies revealed that these cells could exert tumor‐promoting functions. The link between inflammation and cancer has been demonstrated in mouse models of several different cancer types (Greten *et al*, 2004; Pikarsky *et al*, 2004; DeNardo *et al*, 2009; Guerra *et al*, 2011; Jamieson *et al*, 2012; Pyonteck *et al*, 2013; Bald *et al*, 2014; Coffelt *et al*, 2015; Wculek & Malanchi, 2015). For example, in a mouse model of colitis‐associated cancer, genetic depletion of NF‐κB signaling in myeloid immune cells resulted in reduced tumor growth (Greten *et al*, 2004), demonstrating their tumor‐promoting function. Moreover, studies in the *K14‐HPV16* model have shown that mast cells and bone marrow‐derived cells promote squamous skin cancer by activating angiogenesis and by reorganizing stromal architecture via MMP9 (Coussens *et al*, 1999, 2000). Using the same skin cancer model, chronic inflammation was found to promote *de novo* carcinogenesis in a B lymphocyte‐dependent manner (de Visser *et al*, 2005). Since then the tumor‐promoting roles of inflammation‐induced tumor‐associated macrophages (TAMs) (Lin *et al*, 2001; Noy & Pollard, 2014) and neutrophils (Jamieson *et al*, 2012; Bald *et al*, 2014; Coffelt *et al*, 2015, 2016; Wculek & Malanchi, 2015) have been described in several studies. For example, genetic ablation of colony‐stimulating factor 1 (CSF1), an important factor for macrophages, in the *MMTV‐PyMT* breast cancer mouse model delayed progression of mammary tumors to malignancy (Lin *et al*, 2001). Similarly, inhibition of CXCR2, a mediator of neutrophil‐migration, suppressed the formation of intestinal tumors in *APC* ^*min*/+^ mice (Jamieson *et al*, 2012). Together, these studies emphasize that immune cells can act as co‐conspirators in tumor development and progression.

### Deciphering spontaneous metastasis formation

**Figure 4:**
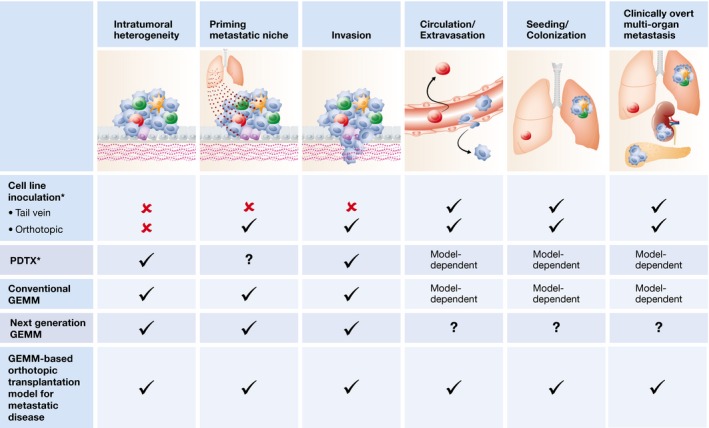
Applications of mouse models in metastasis research This overview summarizes the utility of different preclinical mouse models of experimental and spontaneous metastasis to study the different steps of the metastatic cascade. Conventional GEMMs represent oncomice and TSG knockout mice. Next‐generation GEMMs represent conditional mouse models that are genetically engineered to accurately mimic sporadic human cancer. For some models, the utility for studying specific steps in the metastatic cascade has yet to be determined, as indicated by a question mark. Moreover, several studies have shown that components of the adaptive immune system contribute to the various steps of the metastatic cascade. These aspects cannot be studied in models based on xenografting of human cancer cells or tumor fragments in immunodeficient hosts (indicated by an asterisk). To circumvent this, humanized mice can be used as hosts.

Despite improved cancer treatment options, metastatic disease remains the primary cause of cancer‐related death. The metastatic cascade is a complex multi‐step process managed by a constant crosstalk between cancer cells and their microenvironment (Quail & Joyce, 2013; McAllister & Weinberg, 2014). Most preclinical metastasis research has been performed in cell line inoculation models, which do not recapitulate the metastatic process as it occurs in patients. GEMMs display *de novo* tumor progression and metastasis formation and are therefore indispensable for studying aspects of spontaneous metastasis formation that were unclear in the past (Fig 4). A potential drawback of GEMMs is that mice generally need to be sacrificed due to their primary tumor burden before macroscopic metastases have developed. This problem can be overcome by orthotopic transplantation of GEMM‐derived tumor fragments—which maintain the intratumoral heterogeneity of donor tumors—followed by surgical resection, allowing the development of clinically overt metastatic disease (Doornebal *et al*, 2013).

Several key aspects of metastasis have been discovered in GEMMs. For example, metastasis was originally believed to occur late in tumorigenesis. However, studies in *BALB‐NeuT* and *MMTV‐PyMT* mouse mammary tumor models revealed that transformed cells in early lesions are already capable of disseminating to bone marrow and lungs, and form micro‐metastasis (Hüsemann *et al*, 2008). Similarly, epithelial‐to‐mesenchymal transition (EMT) was thought to play a key role in tumor cell dissemination and metastasis. However, recent studies in GEMMs of pancreatic and breast cancer show that cancer cells retain their epithelial characteristics while colonizing metastatic sites, suggesting that EMT is not essential for metastasis formation in these models (Fischer *et al*, 2015; Zheng *et al*, 2015). Moreover, GEMMs have revolutionized the field by revealing the complex crosstalk between cancer cells and the immune system in metastasis formation. Several laboratories have shown that myeloid immune cells, such as macrophages and neutrophils, play key roles in promoting metastasis formation in different types of cancer (Lin *et al*, 2001; DeNardo *et al*, 2009; Bald *et al*, 2014; Coffelt *et al*, 2015; Wculek & Malanchi, 2015). Recently, we reported a mammary tumor‐induced systemic inflammatory state characterized by IL17‐producing γδ T cells and the subsequent expansion of immunosuppressive neutrophils that drives spontaneous metastasis formation in a GEMM of lobular breast cancer and a GEMM‐based transplantation model for spontaneous metastatic disease (Coffelt *et al*, 2015).

In conclusion, GEMMs have proven indispensable for understanding the complexity of metastasis and have challenged the current dogma that metastasis is a late‐stage cancer cell‐intrinsic process involving EMT. These findings may have important implications for treatment of metastatic cancer patients.

## Applications of GEMMs in translational oncology

**Figure 5:**
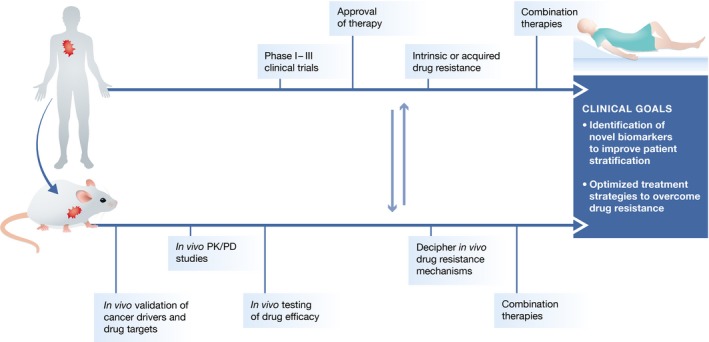
Applications of mouse models in cancer drug development Development of novel treatment strategies in oncology requires preclinical studies in mouse cancer models to identify and validate novel cancer drivers and therapeutic targets, to determine *in vivo* drug pharmacokinetics and pharmacodynamics (PK/PD), and to evaluate *in vivo* anti‐cancer efficacy of novel therapeutics. When promising preclinical results are obtained, the tolerability and anti‐cancer efficacy of these drugs are evaluated in human patients in phase I–III clinical trials. A proportion of patients will show poor response due to intrinsic or acquired resistance, which may be studied mechanistically in preclinical mouse models to identify response biomarkers and combination therapies to prevent or overcome resistance. The close alignment of mouse studies and human clinical trials will lead to better patient stratification, identification of novel biomarkers, and development of optimal combination therapies, culminating in improved cancer patient care.

GEMMs of human cancer have been successfully used to validate candidate drug targets, assess therapy efficacy, and evaluate mechanisms of drug resistance. Since GEMMs develop *de novo* tumors in the context of an intact immune system, they are uniquely suited for investigating the potential of cancer immunotherapy. Close alignment of mouse and human studies can provide a platform that can aid in the development of novel treatment strategies for cancer patients (Fig 5). Below, we discuss how GEMMs can provide clinically relevant information for the design and development of novel anti‐cancer therapies.

### Validation of novel drug targets

Considering that not all cancer genes are essential for maintenance of established tumors, it is important to test whether reactivation of a TSG or down‐regulation of an oncogene results in durable regression of established tumors in a realistic preclinical setting, before drugs against these targets are developed. The relevance of oncogenes for tumor maintenance can be assessed in inducible mouse models in which oncogene expression can be de‐induced once tumors have developed. For example, de‐induction of oncogenic *Pik3ca* ^*H1047R*^ expression in a mouse model of breast cancer caused (partial) tumor regression demonstrating that these tumors are “addicted” to constitutively active PI3K signaling. However, most tumors eventually recurred due to *Met* or *Myc* amplifications, indicating that these genetic lesions may induce resistance to PI3K inhibitors (Liu *et al*, 2011). This example illustrates that preclinical studies in inducible GEMMs are not only useful for validating drug targets but also for identifying mechanisms underlying acquired drug resistance.

TSGs may sometimes also constitute valid drug targets. For example, p53 loss‐of‐function in cancer can result from dominant‐negative or inactivating mutations in the *Trp53* gene or from amplification/overexpression of its specific inhibitors MDM2 and MDM4. Genetic studies in GEMMs with reversible inactivation of p53 have shown that restoration of p53 leads to rapid regression of established tumors (Martins *et al*, 2006; Ventura *et al*, 2007; Xue *et al*, 2007), providing strong rationale for designing anti‐cancer drugs that restore p53 function by inhibiting MDM2 (Vassilev *et al*, 2004) or by restoring wild‐type function to mutant p53 (Bykov *et al*, 2002). Similarly, GEMMs of colorectal cancer with inducible knockdown of APC showed that APC restoration initiates rapid and extensive tumor cell differentiation and sustained regression without relapse, providing *in vivo* validation of the WNT pathway as a therapeutic target for treatment of APC‐mutant colorectal cancers (Dow *et al*, 2015b).

### Unraveling therapy response and resistance

To minimize the risk of failure of novel anti‐cancer therapeutics in clinical trials, preclinical evaluation of response and resistance in robust and predictive *in vivo* models is essential. Therapeutic responses of GEMMs to targeted therapy and conventional chemotherapy are very similar to those of human patients, as was assessed in GEMMs of *Kras*‐mutant lung cancer and pancreatic cancer (Singh *et al*, 2010). However, the differences in drug metabolism between mice and humans have to be taken into consideration. For example, the substrate specificity of the cytochrome P450 enzyme—which is involved in drug metabolism in the liver—is highly variable between species, an issue that could be overcome by humanizing mice (Scheer *et al*, 2012). Hence, preclinical drug efficacy studies in (humanized) GEMMs may advance the development of optimal (combinations of) anti‐cancer drugs to target specific tumors, and the identification of determinants of therapy response that may be used as predictive biomarkers for patient stratification. In addition, GEMMs may be used to identify mechanisms by which therapy‐sensitive tumors acquire drug resistance.

A clear example of a preclinical GEMM that has provided mechanistic insight into therapy response and resistance of *BRCA1*‐mutated breast cancer is the *K14cre;Brca1* ^*F*/*F*^ *;Trp53* ^*F*/*F*^ (KB1P) mouse model. KB1P mice develop mammary tumors that mimic the histopathological features of human *BRCA1*‐mutated breast cancers as well as their hypersensitivity to platinum drugs and PARP inhibitors (Rottenberg *et al*, 2007, 2008). Clinical trials evaluated the PARP inhibitor olaparib for the treatment of ovarian, breast, and colorectal cancer (Lee *et al*, 2014). While olaparib did not seem promising in this diverse group of cancer patients, it did show significant responses in *BRCA1*‐mutation carriers, due to the synthetic lethal combination of PARP inhibition and BRCA1‐deficiency (Ledermann *et al*, 2012, 2014). *BRCA1*‐mutant cells are more vulnerable to PARP inhibition because the single‐strand DNA breaks induced by PARP inhibition, lead to double‐strand breaks during replication, which cannot be repaired by BRCA1‐deficient cells due to lack of homologous recombination. Based on promising results obtained in clinical trials (Ledermann *et al*, 2012, 2014), olaparib (trade name LynParza) was approved by the FDA in December 2014 for the treatment of patients with advanced *BRCA1*/*2*‐mutated ovarian cancer. Despite the good response of *BRCA1*/*2*‐mutated cancers to olaparib, acquired resistance is observed both in patients and GEMMs. Preclinical studies in KB1P mice revealed several mechanisms of resistance, such as elevated levels of drug efflux transporters and restoration of homologous recombination (Rottenberg *et al*, 2008; Jaspers *et al*, 2013; Henneman *et al*, 2015; Xu *et al*, 2015). These studies could aid in understanding clinical resistance and in designing improved treatment strategies for olaparib‐resistant patients in the clinic.

It is becoming clear that therapy response and resistance is not only influenced by tumor cell‐intrinsic factors but also by stromal factors such as fibroblasts and immune cells (Farmer *et al*, 2009; DeNardo *et al*, 2011; Shree *et al*, 2011; Acharyya *et al*, 2012; Nakasone *et al*, 2012; Boelens *et al*, 2014). This is illustrated by tumor intervention studies in a GEMM of PDAC, which showed that therapeutic inhibition of paracrine Sonic Hedgehog (SHH) signaling reduced desmoplastic tumor stroma and increased tumor vasculature, resulting in enhanced delivery of gemcitabine to tumors (Olive *et al*, 2009). However, the concept of targeting tumor stroma in PDAC has recently been challenged by two studies showing that stromal factors may suppress rather than promote PDAC growth, possibly by restraining tumor angiogenesis (Özdemir *et al*, 2014; Rhim *et al*, 2014). Together, these studies demonstrate that the contribution of the tumor microenvironment to therapy resistance may be more profound, but also more complex, than previously anticipated.

### Cancer immunotherapy

Over the past decades, mechanistic insights into immune responses have culminated in therapeutic strategies that harness the patient's immune system to attack cancer. Recent clinical trials in patients with advanced melanoma and lung cancer confirm the remarkable potential of immune checkpoint blockade, including anti‐CTLA‐4 and anti‐PD‐1, to enhance effective anti‐tumor immunity and to improve survival in a proportion of the patients (Hodi *et al*, 2010; Topalian *et al*, 2012, 1). The basis of these clinical trials comes from several decades of fundamental research in experimental mouse models that have revealed the importance of CTLA‐4 and PD‐1 in restraining immune responses, as most clearly illustrated by the severe spontaneous autoimmunity phenotype in CTLA‐4‐deficient (Waterhouse *et al*, 1995) and to a milder extent in PD‐1‐deficient mice (Nishimura *et al*, 1999, 2001). CTLA‐4 blockade in mice bearing inoculated tumors enhances anti‐tumor T‐cell responses resulting in tumor rejection (Leach *et al*, 1996), illustrating that releasing the brake on T cells might be an interesting strategy to combat cancer. Nevertheless, a substantial proportion of patients do not respond to immunotherapy, and the current challenge is to understand why.

Currently, the majority of immunological studies are performed in tumor transplantation models, but we foresee a growing role for GEMMs. Several studies in GEMMs illustrate that during *de novo* carcinogenesis, T cells fail to respond due to tumor‐induced tolerance mechanisms (Willimsky & Blankenstein, 2005; Garbe *et al*, 2006; DuPage *et al*, 2011). Strikingly, transplantation of GEMM‐derived tumor cells in immunodeficient mice resulted in rapid tumor growth, while wild‐type mice rejected these tumors (Willimsky & Blankenstein, 2005; Garbe *et al*, 2006; DuPage *et al*, 2011), demonstrating that the cancer cells did not lose their immunogenicity and T cells are still able to recognize and attack them; however, they fail to do so in a host bearing *de novo* tumors.

Tumors are often characterized by chronic inflammation, which induces local and systemic immunosuppression that is unfavorable for T cells to perform their effector function (Mitchem *et al*, 2013; Ruffell *et al*, 2014; Coffelt *et al*, 2015). Moreover, tumors often show dysfunctional dendritic cells, which results in impaired T‐cell priming. For example, *MMTV‐PyMT* mammary tumors display dendritic cells that are potent activators of anti‐tumor T cells, but these cells are outcompeted by the overabundant presence of macrophages preventing proper T‐cell activation (Engelhardt *et al*, 2012; Broz *et al*, 2014). Recent studies have demonstrated that boosting dendritic cell function (Broz *et al*, 2014; Ruffell *et al*, 2014; Salmon *et al*, 2016; Sánchez‐Paulete *et al*, 2016) or blocking myeloid cell‐induced immunosuppression (Highfill *et al*, 2014; Zhu *et al*, 2014) improves the anti‐tumor efficacy of immune checkpoint blockade. Thus, patients that show resistance to T‐cell boosting immunotherapy might show improved clinical benefit when treatment is combined with compounds that either target immunosuppression or enhance T‐cell priming.

Immunotherapy studies in GEMMs require a different approach compared to inoculation models. Considering that tumors in GEMMs develop *de novo,* individual mice—like patients—have a unique set of tumor antigens. Consequently, heterogeneous responses are expected, which warrants identification of molecular differences between responsive and non‐responsive tumors, and may yield biomarkers that can predict clinical benefit. However, for most GEMM‐derived tumors, the identity of expressed tumor antigens that could be recognized by T cells is unknown. To circumvent this, clinically relevant tumor antigens could be introduced by genetic engineering to allow tracking of tumor‐specific T‐cell responses. For example, the introduction of tumor‐specific antigens in GEMMs with low immunogenic tumors, such as sarcomas and lung cancer, increased the immunogenicity of these tumors and resulted in a potent, but transient, anti‐tumor T‐cell response (DuPage *et al*, 2011, 2012). The initial anti‐tumor T‐cell response was quickly followed by regulatory T‐cell‐mediated immunosuppression (Joshi *et al*, 2015). Thus, these models can aid ongoing and future research to unravel the complex mechanisms underlying immune evasion (DuPage & Jacks, 2013), and may ultimately lead to novel (combination) strategies to improve cancer immunotherapy.

### Co‐clinical trials in GEMMs

Recently, a “co‐clinical trial” paradigm has been developed in which preclinical trials in GEMMs are run in parallel with human clinical trials to predict therapeutic response (Clohessy & Pandolfi, 2015). This strategy was successfully used to identify genetic determinants of androgen deprivation resistance in prostate cancer, as well as novel combination therapies to overcome castration resistance (Lunardi *et al*, 2013). Similarly, co‐clinical trials in GEMMs of NSCLC showed that *Kras*/*Lkb1*‐mutant lung tumors are more resistant to combination therapy with docetaxel and the MEK inhibitor selumetinib than *Kras*‐ or *Kras*/*p53*‐mutant tumors, highlighting *LKB1* as a potential determinant of resistance in clinical trials with this combination therapy (Chen *et al*, 2012). These studies demonstrate that preclinical efficacy studies in GEMMs of human cancer may identify novel biomarkers and (combination) therapies that can be validated in concurrent human clinical trials or used to optimize the design of future trials.

## Concluding remarks and future perspectives

Many anti‐cancer drugs in clinical trials do not live up to the high expectations raised by preceding preclinical studies. How can we improve the predictive power of preclinical studies in the oncology arena? Most importantly, the preclinical tumor model of choice should reflect the human disease as faithfully as possible. To achieve this, it is important that preclinical mouse models capture both the intrinsic and extrinsic properties of cancer. First, preclinical models should contain the patient‐specific mutations that initiated the malignancy, and harbor the genetic variation as seen in patient populations. In addition, *de novo* tumorigenesis should occur in the natural microenvironment reflecting the crosstalk of cancer cells with the tumor microenvironment (including infiltrating immune cells, fibroblasts, and the lymphatic and blood vasculature) as observed in human cancer. It is also important to realize that the majority of patients who are enrolled in clinical trials have already developed advanced metastatic disease. Preclinical drug efficacy studies should therefore be preferably performed in mice that reflect the disease stage of the patients for which the therapy is intended. On a similar note, patients enrolled in clinical trials are frequently heavily pre‐treated, which is likely to negatively affect therapy outcome. Preclinical studies performed in treatment‐naive animals may thus overestimate therapy efficacy. On the other hand, treatments that are unsuccessful in heavily pre‐treated patients with advanced disease might still be beneficial for treatment‐naive patients with less advanced disease.

Current advances in genetic engineering allow for fast‐track generation and genetic fine‐tuning of mouse models that develop *de novo* cancer, which incorporates both cancer cell‐intrinsic and cell‐extrinsic properties of specific patient cohorts. We anticipate that these next‐generation GEMMs and GEMM‐based orthotopic transplantation models for spontaneous metastatic disease are currently the best available models to faithfully recapitulate human cancer. These models provide valuable tools to study the mechanisms underlying complex processes such as cancer initiation, organ‐specific metastasis formation, and the involvement of tumor microenvironment. But, more importantly for cancer patients, these models will provide better insights into (immune) therapy responsiveness and resistance, and disease recurrence. It is expected that preclinical efficacy studies with novel anti‐cancer drugs in next‐generation GEMMs will provide enhanced predictivity for their clinical efficacy, and thus accelerate the design and clinical implementation of novel anti‐cancer strategies that will improve cancer patient care.

## Conflict of interest

The authors declare that they have no conflict of interest.

**Pending issues**

- Understanding of tumor cell‐intrinsic and cell‐extrinsic mechanisms underlying cancer and metastasis development, and therapy resistance.
- Development of multidisciplinary therapeutic strategies including conventional anti‐cancer drugs and immunotherapy to successfully fight disseminated cancer.
- Reduction in time and costs to generate next‐generation genetically engineered mouse models that closely recapitulate human cancer.
- Close alignment of preclinical mouse studies and human clinical trials to improve cancer patient care.
